# Strength of Structural and Functional Frontostriatal Connectivity Predicts Self-Control in the Healthy Elderly

**DOI:** 10.3389/fnagi.2016.00307

**Published:** 2016-12-23

**Authors:** Jürgen Hänggi, Corinna Lohrey, Reinhard Drobetz, Hansruedi Baetschmann, Simon Forstmeier, Andreas Maercker, Lutz Jäncke

**Affiliations:** ^1^Division of Neuropsychology, Department of Psychology, University of ZurichZurich, Switzerland; ^2^Division of Psychopathology and Clinical Intervention, Department of Psychology, University of ZurichZurich, Switzerland; ^3^Developmental Psychology, Department of Education Studies and Psychology, University of SiegenSiegen, Germany; ^4^University Research Priority Program (UFSP), Dynamic of Healthy Aging, University of ZurichZurich, Switzerland; ^5^International Normal Aging and Plasticity Imaging Center, University of ZurichZurich, Switzerland; ^6^Center for Integrative Human Physiology, University of ZurichZurich, Switzerland

**Keywords:** delay of gratification and delay discounting, putamen, caudate and accumbens, dorsolateral and ventrolateral prefrontal cortex, healthy aging and wellbeing, diffusion tensor imaging, resting state functional magnetic resonance imaging

## Abstract

Self-regulation refers to the successful use of executive functions and initiation of top-down processes to control one's thoughts, behavior, and emotions, and it is crucial to perform self-control. Self-control is needed to overcome impulses and can be assessed by delay of gratification (DoG) and delay discounting (DD) paradigms. In children/adolescents, good DoG/DD ability depends on the maturity of frontostriatal connectivity, and its decline in strength with advancing age might adversely affect self-control because prefrontal brain regions are more prone to normal age-related atrophy than other regions. Here, we aimed at highlighting the relationship between frontostriatal connectivity strength and DoG performance in advanced age. We recruited 40 healthy elderly individuals (mean age 74.0 ± 7.7 years) and assessed the DoG ability using the German version of the DoG test for adults in addition to the delay discounting (DD) paradigm. Based on diffusion-weighted and resting-state functional magnetic resonance imaging data, respectively, the structural and functional whole-brain connectome were reconstructed based on 90 different brain regions of interest in addition to a 12-node frontostriatal DoG-specific network and the resulting connectivity matrices were subjected to network-based statistics. The 90-nodes whole-brain connectome analyses revealed subnetworks significantly associated with DoG and DD with a preponderance of frontostriatal nodes involved suggesting a high specificity of the findings. Structural and functional connectivity strengths between the putamen, caudate nucleus, and nucleus accumbens on the one hand and orbitofrontal, dorsal, and ventral lateral prefrontal cortices on the other hand showed strong positive correlations with DoG and negative correlations with DD corrected for age, sex, intracranial volume, and head motion parameters. These associations cannot be explained by differences in impulsivity and executive functioning. This pattern of correlations between structural or functional frontostriatal connectivity strength and self-control suggests that, in addition to the importance of the frontostriatal nodes itself, the structural and functional properties of different connections within the frontostriatal network are crucial for self-controlled behaviors in the healthy elderly. Because high DoG/low DD is a significant predictor of willpower and wellbeing in the elderly population, interventions aiming at strengthening frontostriatal connectivity to strengthen self-controlled behavior are needed in the future.

## Introduction

From early childhood, through adulthood, up to high age, people must continuously make decisions between immediate and delayed rewards. The concept of delay of gratification (DoG), established by Walter Mischel (Mischel et al., 1989), describes the voluntary postponement of smaller immediate benefits for better later rewards. Instead of taking one marshmallow now, holding out for two or three marshmallows later is an example of DoG. Delay discounting (DD), also called temporal discounting, has been defined as a reduction in the subjective value of a reward as later as the reward is delivered (Kirby and Marakovic, 1996; Klapproth, 2008). Low DD and high DoG reflect better self-control; therefore, the two measures are inversely correlated (Forstmeier et al., 2011; Drobetz et al., 2014).

To the best of our knowledge, no functional imaging study has been conducted in the healthy elderly using the concept of DoG. However, in a recently published structural MRI (sMRI) study of the specific behavioral construct of DoG, Drobetz and colleagues used surface-based morphometry and subcortical segmentation procedures and showed that DoG and DD performance are significantly associated with local cortical thickness and surface area in the dorsolateral prefrontal (DLPFC), ventrolateral prefrontal (VLPFC), orbitofrontal (OFC), and anterior cingulate (ACC) cortices on one hand and with the volume of the caudate nucleus and nucleus accumbens on the other hand (Drobetz et al., 2014).

Three years ago, positive correlations between DD and functional connectivity in the right ventromedial prefrontal cortex (VMPFC) and middle temporal regions in older adults were demonstrated using resting-state functional MRI (rsfMRI) (Han et al., 2013). However, in that study, DD was also significantly negatively correlated with right cerebellar and parahippocampal regions, which are more difficult to relate to the concepts of self-control and willpower (Han et al., 2013). Behavioral investigations using the concept of DD have reported inconsistent findings with respect to age-related differences in DD. The key concepts of DoG, DD, self-regulation, self-control, and impulsivity are interrelated. DD, impulsivity, and their related constructs are associated with the DLPFC, OFC, ACC, and the striatum as has been repeatedly reported in neuroimaging studies (McClure et al., 2004, 2007; Bjork et al., 2009; Matsuo et al., 2009).

It has been suggested that there exist two separate brain systems that modulate immediate and delayed reward decisions differently. The “impatient emotional system” (ventral striatum, VLPFC, and posterior cingulate cortex) values immediate rewards, while the “rational cognitive system” (DLPFC and posterior parietal cortex) values both types of reward, the immediate and delayed one (McClure et al., 2004, 2007). However, the idea of two separate brain systems being involved in DD has been challenged by other studies mainly because the analysis of the delay discounting rate k is typically associated with hyperbolic models of DD and are usually interpreted as representing a single-system. In the present study, however, we aimed at elucidating neural correlates associated with DoG and/or DD. Whether the concept of DoG or DD is more adequately described in terms of a single or rather a dual brain system theory is not focus of our work and hence not addressed in the present study. It might be the case that a dual brain system theory as described above is more adequate for the concept of DoG, whereas a single brain system theory is more adequate for the concept of DD. Whether hyperbolic models typically used to estimate the DD rate k are compatible with a dual system theory in the brain remains to be shown in future investigations. Behaviorally, it has also been shown, for instance, that there is a lower tendency to discount the future with advancing age, an effect mainly driven by age differences in affective responses and mental health rather than cognitive or demographic variables (Löckenhoff et al., 2011) suggesting that only the “impatient emotional system” might be affected in the elderly.

In general, decisions between delayed and immediate rewards involve the right DLPFC, right VLPFC, left and right parietal cortices surrounding the intraparietal sulcus (McClure et al., 2004), VMPFC (Pedroni et al., 2011) and the right lateral OFC, independently of whether delayed and immediate rewards are chosen (McClure et al., 2004). It has also been shown that the subjective value of immediate and delayed rewards are encoded in the posterior cingulate cortex, in addition to the ventral striatum and the medial prefrontal cortex (Kable and Glimcher, 2007, 2010). In the context of aging, it has been revealed that reduced ventral striatal activations to immediate reward are associated with less impulsive decision-making in older adults suggesting reduced sensitivity of striatal areas to reward (Eppinger et al., 2012). Age-related reductions in brain activity in the VMPFC during learning from reward and a decreased responsivity of the ventral part of the striatum in response to reward prediction errors during learning in the elderly compared with younger adults have been reported as well (Eppinger et al., 2013). For a broader view of decision making in the aging brain and with respect to age-related changes in affective and motivational circuits the reader is referred to an excellent review (Samanez-Larkin and Knutson, 2015).

Normal age-related structural and functional connectivity alterations independent of decision making are not the focus of the present study and have already been reported and reviewed in the literature elsewhere (Andrews-Hanna et al., 2007; Ferreira and Busatto, 2013; Antonenko and Flöel, 2014; Bennett and Madden, 2014; Dennis and Thompson, 2014; Sexton et al., 2014; Sala-Llonch et al., 2015; Ferreira et al., 2016; Fjell et al., 2016). In summary, these studies showed that there is a normal age-related decline in structural as well as functional connectivity strength that is not uniformly distributed throughout the brain, i.e., the magnitude of connectivity reductions differs between brain regions.

In the present combined structural and functional MRI-based connectivity study, we mainly aimed at identifying structural and functional connections, the strengths of which are associated with the DoG ability in healthy elderly subjects, but we also investigated connectivity correlates associated with the more famous concept of DD. The study is motivated by the fact that structural and functional connectivity declines with advancing age are not uniformly distributed, and indeed the prefrontal cortex is more prone to normal age-related atrophy compared with other brain regions (Fjell et al., 2009), implying the potential to adversely affect frontostriatal connectivity, which might in turn result in poorer DoG and DD performance in the healthy elderly. Therefore, we investigated connectome-related structural and functional correlates associated with DoG (or DD) in healthy elderly individuals to determine whether normal age-related declines in structural and functional frontostriatal connectivity strength adversely affect the ability of DoG and DD.

## Materials and methods

### Subjects and study design

Forty-two native Swiss-German- or German-speaking elderly people (14 men, 26 women) with a mean age of 74.0 ± 7.7 years (range: 63–93 years) and a mean of 12.8 ± 2.3 educational/academic years (range: 9–20 years) were recruited from a behavioral DoG study of a large sample of 120 healthy, cognitively unimpaired elderlies (Forstmeier et al., 2011). A lack of cognitive impairment as indexed by a score of ≥26 on the Mini Mental State Examination (MMSE; Folstein et al., 1975) and age of ≥60 years were the inclusion criteria for the current neuroimaging study. Current mental health disorders, neurological disorders, and history of stroke or dementia were the exclusion criteria. One participant reported a history of apoplexy and one was taking drugs for attention deficit hyperactivity disorder and therefore these two subjects had to be excluded from the current investigation. An additional subject was excluded for the rsfMRI analysis only because the rsfMRI scans did not cover the whole brain. All participants provided written informed consent and were paid CHF 100 for their willingness to participate. The study protocol was conducted in accordance with the Declaration of Helsinki and approved by the ethics committee of the canton of Zurich, Switzerland.

### Behavioral measures

We applied the German version of the DoG test for adults (BAT-E) (Forstmeier et al., 2011), but in addition we also used the German version of the more known (DD) paradigm (Kirby and Marakovic, 1996) that is related to DoG (Forstmeier and Maercker, 2011). The DoG paradigm (BAT-E) has been described in more detail elsewhere (Forstmeier et al., 2011), but is reiterated here in exactly the same wording as formerly already published by our group (Drobetz et al., 2014). “The test requires participants to make 18 choices between immediate smaller and delayed larger primary and secondary reinforcers: snacks, hypothetical money, real money, and magazines. In the snacks subscale, the experimenter asks the participant to decide between one piece of chocolate immediately and two pieces in 2 h. In the real money subscale, the participant must decide between CHF 8 now or CHF 10 in 1 month. In the hypothetical money subscale, the participant must choose between smaller amounts of hypothetical money now and larger amounts of hypothetical money in 1 month. In the magazines subscale, the participant must choose either one real magazine now or two real magazines in 1 month. The snacks and hypothetical money subscales consist of eight items, the real money and magazine subscale consist of two items—in sum, the DoG-A has 18 items. Whereas the snacks and hypothetical money subscores range from 0 to 8, the real money and magazines subscores are dichotomous (immediate real money/ magazine now vs. delayed real money/two magazines). In each of the 18 items, the participant received one point when he/she chose the delayed reward and zero points when he/she took the immediate reward. We used the DoG-A score, the sum of all four subscales, as a composite measure ranging from 0 (nondelayer in all four subscales) to 4 (delayer in all four subscales)” (Drobetz et al., 2014).

Impulsivity has been measured with the German version of Barratt impulsiveness scale (BIS-11; Patton et al., 1995; Preuss et al., 2008) and executive functions were assessed by three different cognitive tests: Trail making test—part B (Reitan, 1958), digit span backward test (Wechsler, 1997), and the Stroop color-word test (Stroop, 1935). For executive functioning, we calculated composite scores (z-scores) for all individuals based on the three executive function tests applied, i.e., the trail making test—part B, digit span backward test and the Stroop color-word test.

### Magnetic resonance imaging data acquisition

We used a 3.0 Tesla Philips Achieva whole-body scanner (Philips Medical Systems, Best, The Netherlands) to acquire the MRI data. The MRI scanner was equipped with a common eight-element head coil array capable of sensitivity encoding (SENSE) and a transmit-receive body coil.

Two T1-weighted images were acquired, in addition to the one resting-state functional MRI (rsfMRI) and one diffusion-weighted imaging sequence that have been applied for each subject under investigation.

For all 40 participants, a volumetric three dimensional T1-weighted gradient echo sequence named fast field echo has been applied twice resulting in two T1-weighted MRI scans. With a spatial resolution of 0.94 × 0.94 × 1.00 mm^3^ (matrix 256 × 256 pixels, 160 slices), the slices were acquired in the sagittal plane. Other technical parameters of the T1-weighted sequence were: field of view FOV = 240 × 240 mm^2^; echo time TE = 3.7 ms; repetition time TR = 8.06 ms; flip angle = 8°; and SENSE factor = 2.1. Scan time was about 8 min per scan. To exclude any T2-sensitive tissue anomalies, T2-weighted MRI scans were acquired as well.

Subjects rested quietly with closed eyes in the scanner while rsfMRI time series have been acquired. Participants were instructed to let their mind wander and to think of nothing in particular. Images of rsfMRI were acquired in the transversal plane using a spin-echo echo-planar imaging (EPI) sequence with a measured spatial resolution of 2.5 × 2.5 × 4.0 mm^3^ (reconstructed 1.72 × 1.72 × 4.0 mm^3^). Imaging parameters were: TR = 4.0 s (interleaved slice acquisition starting with slice one); TE = 35 ms; FOV = 220 × 220 mm^2^; matrix 112 × 112 pixels; slice thickness = 4 mm; number of slices = 40; flip angle = 78°; SENSE factor = 1.8. The rsfMRI sequence lasted about 10 min (corresponding to 150 brain volumes).

Subsequently, a diffusion-weighted sequence with a spatial resolution of 2 × 2 × 2 mm^3^ (matrix: 112 × 112 pixels, 75 slices in transversal plane) was applied. Diffusion was measured along 32 non-collinear directions (*b* = 1000 s/mm^2^) preceded by a non-diffusion-weighted volume (reference volume, *b* = 0 s/mm^2^). Further imaging parameters were: FOV = 224 × 224 mm^2^; TE = 55.0 ms; TR = 13.010 s; flip-angle = 90°; SENSE factor = 2.1. Scan time was about 8 min 42 s. T1-weighted, DTI, and resting state fMRI images were acquired in the same session.

### Data preprocessing for structural connectome analyses

The FMRIB software library tools (FSL, version 5.0.7; http://www.fmrib.ox.ac.uk/fsl/; Smith et al., 2004) such as the FDT (FMRIB diffusion toolbox; version 3.0; Behrens et al., 2003) has been used for preprocessing of the diffusion-weighted MRI data. The Diffusion Toolkit (DTK, version 0.6.3) and TrackVis software (version 0.6.0.1; http://trackvis.org/; Park et al., 2009) has been applied to achieve deterministic fiber tractography. The connectivity matrix has been computed in MATLAB software (version 2013b; http://www.mathworks.com/index.html).

To construct the connectivity matrix of the white matter pathways, the following fully automated preprocessing steps were realized: (1) In a first step, a binary brain mask was created using FSL's brain extraction tool. This mask was used in later steps to exclude non-brain tissue. (2) Eddy current distortions and head movements were corrected using the EDDY_CORRECT tool of FDT. (3) Extraction of the mean translation and rotation values according to work done by Yendiki et al. (2014) for later use of these measures as a covariate of no interest in the regressions. This has been achieved by using the TRACULA tool (Yendiki et al., 2011) (https://surfer.nmr.mgh.harvard.edu/fswiki/Tracula) implemented in the FreeSurfer software suite (http://surfer.nmr.mgh.harvard.edu/fswiki). (4) Diffusion gradients were adjusted for rotations introduced by the eddy current and head movement corrections. (5) The preprocessed DTI data were then subjected to the DTK to compute voxel-wise diffusion tensors and to construct the (principal) eigenvector and eigenvalue maps as well as a map of fractional anisotropy (FA). (6) Deterministic tractography was conducted in TrackVis using the “brute force” approach with an interpolated streamline tracking algorithm. Twenty streamlines per voxel were propagated (using the -rseed option in DTK with value 20) and fiber tracking algorithm terminated if the voxel's FA value drop below 0.10 or if the streamline's turning angle between two consecutive voxels increase over 45°. This results in a connectome of the whole-brain comprised by about 2–3 million of streamlines including connections to the cerebellum and subcortical pathways. (7) The individual FA image was mapped onto the FMRIB58-FA reference brain, which is already in the MNI152 standard space, using FSL's linear image registration tool (abbreviated FLIRT). The obtained transformations were stored for later use. (8) We then applied these transformations to the streamlines derived from step 6 to map the streamlines into the MNI152 standard space. (9) To count the number of streamlines between each pair of ROIs we used the automated anatomical labeling (AAL) atlas (Tzourio-Mazoyer et al., 2002), the ROIs of which are already located in MNI152 standard space. Covering the entire neocortex (78 cortical ROIs), the subcortical structures amygdala, hippocampus, thalamus, caudate, putamen, and pallidum (12 subcortical ROIs) as well as 26 cerebellar ROIs, the AAL atlas consists of 116 ROIs in total. However, the 26 cerebellar ROI were excluded because we did not expect any correlates of DoG od DD in the cerebellum, which however was not entirely covered by MRI in all subjects. (10) Through the brainstem running streamlines and that shorter than 5 mm in length were removed. Self-loops, i.e., streamlines that make connections within a ROI were discarded. Using MATLAB scripts written by Zalesky (Zalesky et al., 2010), we counted the number of the remaining streamlines between any pair of ROIs resulting in an undirected, weighted, 90 × 90 connectivity matrix for each individual participant. Hence, the strength of a structural connection between two ROIs was operationalized by the number of reconstructed streamlines. (11) The undirected, weighted 90 × 90-node connectivity matrices were then subjected to a network-based statistical analysis (see below). We also investigated a smaller and more DoG-specific network comprised by only frontostriatal brain regions that were derived from a subcortical brain atlas and from a surface-based morphometric imaging study (Drobetz et al., 2014) that investigated structural gray matter correlates of DoG in the same participants as investigated in the present study.

### Data preprocessing for functional connectome analyses

DPARSFA toolbox (version 3.1) that is part of DPABI (version 1.2, http://rfmri.org/dpabi; Chao-Gan and Yu-Feng, 2010) and depends on functions of statistical parametric mapping software (SPM, version 8; www.fil.ion.ucl.ac.uk/spm/software/spm8) has been used for functional MRI data preprocessing. Following preprocessing steps has been performed: (1) Coregistration of the T1-weighted image onto the functional images. (2) slice timing correction, (3) realignment combined with the extraction of the frame-wise displacement parameters according work by Power and colleagues (Power et al., 2012, 2014) for later use as a covariate of no interest in the regressions, (4) estimation of linear and non-linear normalization of the T1-weighted MRI image using the unified segmentation approach as implemented in SPM8, (5) estimated transformations were then applied to the functional images, (6) voxel re-sampling to 2 × 2 × 2 mm^3^, (7) smoothing with a Gaussian kernel of 6 mm full width at half maximum, (8) detrending, (9) data filtering in the range 0.01 < f <0.1 Hz, and (10) regressing out the variance of nine nuisance covariates, i.e., the six parameters from head motion correction (three translation and three rotation parameters) as well as the global mean signal, white matter signal, and cerebrospinal fluid signal. Although, there is an ongoing dispute about whether regressing out the global mean signal in rsfMRI data analyses is beneficial or affects data detrimentally (Wong et al., 2012; Qing et al., 2015; Yeh et al., 2015), we applied global mean signal regression because it has also been shown that the global mean signal regression is very effective in controlling movement-related artifacts in functional connectivity measures (Power et al., 2012, 2014; Satterthwaite et al., 2012, 2013; Van Dijk et al., 2012; Yan et al., 2013).

### Regions of interest definition for the delay of gratification-specific frontostriatal network

In addition to the 90-node whole-brain network analyses, a 12-node frontostriatal network including DoG-related brain regions has been constructed. This network was comprised by the subcortical structures putamen, caudate nucleus, and nucleus accumbens and the cortical structures OFC as well as DLPFC and VLPFC. The subcortical structures were derived from the Harvard-Oxford subcortical atlas as implemented in the FSL viewer (version 3.0, http://fsl.fmrib.ox.ac.uk/fsl/fslwiki/Atlases), whereas the cortical regions were derived from a former study of our group showing that regional cortical thickness and cortical surface area in the OFC, DLPFC, and VLPFC is related to DoG performance the same participants as the ones investigated in the present study (Drobetz et al., 2014), which also reported associations between DoG and DD performance and the volume of the caudate nucleus and nucleus accumbens. This DoG-specific 12-node network was used for both structural and functional network analyses.

### Network-based statistical analyses

The network-based statistical analyses of the structural (DTI) and functional (rsfMRI) connectivity matrices have been performed using the network-based statistic (NBS) toolbox (Zalesky et al., 2010) (https://www.nitrc.org/projects/nbs/) running in MATLAB (version R2013b). When mass-univariate testing is performed at every connection comprising the graph (network), NBS can be used to control the family-wise error rate. NBS is based on common principles underpinning other traditional cluster-based thresholding procedures applied onto statistical parametric maps and it exploits the extent to which the connections comprising the effect of interest are interconnected (Zalesky et al., 2010).

Based on the general linear model approach, we used the *t*-test module in NBS to correlate DoG and DD performance with the number of reconstructed streamlines of each connection (structural connectome) or the Fisher's z-transformed correlation coefficient between two brain regions (functional connectome) of the 90- and 12-node networks while simultaneously controlling for the influences of age, sex, and total intracranial volume. In addition to these three covariates, we also used the frame-wise displacement value encompassing translational and rotational motion parameters (Power et al., 2012, 2014) derived from the volume-by-volume realignment as a covariate of no interest in the functional connectivity analyses and the volume-by-volume realignment parameters (Yendiki et al., 2014) in the structural connectivity analyses in order to account for residual head motion-related influences in the connectivity measures. These partial correlations were done with the *component extent* option in NBS that is suited for detecting an effect of interest that is relatively weak at one connections, but extends to encompass a lot of connections (Zalesky et al., 2010). In all analyses, we simultaneously applied multiple comparisons correction procedures using 5000 permutations at *p* < 0.05. *P*-values of the network-based statistical analyses are reported one-tailed due to the directed contrasts.

It is important to note that the main statistical threshold (alpha error probability) was set at *p* = 0.05 corrected for multiple comparison using 5000 permutations for all network analyses reported in the present manuscript. The t-thresholds reported (see Results Section below) do not represent the alpha error probabilities. These t-thresholds are set (also called sensitivity) thresholds (Zalesky et al., 2010) and are used to determine which edges of the connectivity matrix form the largest subnetwork, which is then subjected to the permutation statistic. These sensitivity thresholds must be determined by exploration and are therefore chosen in an arbitrary way. However, this does not affect the false positive rate of the actual permutation statistic of the alpha error probability.

Two different thresholded subnetworks are reported per behavioral measure (DoG or DD), i.e., one subnetwork with many connections (more liberal set threshold) and one subnetwork with only few connections (more conservative set threshold) by applying two different set (sensitivity) thresholds (operationalized as *t*-values, called set *t*-value in the rest of the manuscript; Zalesky et al., 2010). The more conservative set threshold has been chosen to show the specificity of the frontostriatal connections within the subnetworks. However, for both differently thresholded subnetworks alpha error probability was set at *p* = 0.05 while simultaneously correcting for multiple comparison using 5000 permutations of the behavioral measure across subjects. Positive as well as negative associations have been tested separately. Significant subnetworks are visualized using BrainNet Viewer software (https://www.nitrc.org/projects/bnv/; Xia et al., 2013). Due to reasons of completeness and because of literature cited in the Discussion Section, structural and functional connectivity strength have also been correlated with impulsivity and executive functioning using the same covariates of no interest as used for DoG and DD. However, impulsivity and executive functioning have mainly been investigated to show the specificity of DoG and DD ability with respect to frontostriatal connectivity strength, i.e., these associations represent negative control conditions and hence no hypotheses were posed a priori.

### Other statistical analyses

Correlations between behavioral measures and the movement parameters of the functional and structural MRI data have been performed using IBM SPSS Statistics software (version 23.0, http://www-01.ibm.com/software/analytics/spss/). One-tailed *p*-values are reported for the network analyses because t-contrasts in NBS are directed and two-tailed *p*-values are reported for all other statistical tests. If exceptions are made, they are explicitly indicated.

## Results

### Demographic, cognitive, and global brain measures

The mean values and standard deviations of the demographic, cognitive as well as global brain measures of the 40 subjects under investigation are summarized in Table 1.

**Table 1:** **Demographic and behavioral characteristics as well as global brain measures of the elderly subjects under investigation (*n* = 40)**.

**Variable**	**Mean ± SD (range)**
Age	73.7 ± 7.7 (63–93)
Gender	26 female (65.0%)
Education (years)	12.8 ± 2.3 (9–20)
Verbal intelligence	34.4 ± 2.7 (27–40)
Number of right handed participants	25 (62.5%)
Mini mental state examination	29.3 ± 1.0 (26–30)
DoG-A score	1.82 ± 1.39 (0–4)
Delay discounting (k)	−4.5 ± 1.8 (−8.7 to −1.4)
Executive functioning	0.00 ± 0.5 (−1.1 to −1.3)
Impulsivity	60.1 ± 7.0 (44–78)
Intracranial volume (cm^3^)	1348.7 ± 240.6 (960.4–1986.2)
Total cortical white matter volume (cm^3^)	443.0 ± 60.4 (322.5–600.5)
Total cortical gray matter volume (cm^3^)	405.6 ± 40.9 (334.8–509.0)
Total cortical surface area (cm^2^)	1535.8 ± 166.9 (1251.2–1980.4)
Mean cortical thickness (mm)	2.407 ± 0.113 (2.175–2.614)

*DoG-A score, Delay of Gratification Test for Adults–composite score (Forstmeier et al., 2011). Verbal intelligence was measured by the German vocabulary test (Schmidt and Metzler, 1992). Impulsivity was measured by the Barratt Impulsiveness Scale (Patton et al., 1995; Preuss et al., 2008). Executive function refers to a composite measure (z-scores) of the following tests of executive functions: Trail Making Test—Part B (Reitan, 1958), Digit Span Backward test (Wechsler, 1997), and Stroop Color-Word Test (Stroop, 1992)*.

As already reported elsewhere (Drobetz et al., 2014), the total DoG score of the BAT-E and the global DD rate (k) of the delay-discounting test are inversely correlated (*r* = −0.33, *p* = 0.039). The total DoG score did neither statistically significantly correlate with impulsivity (*r* = 0.08, *p* = 0.61) nor with executive functions (*r* = 0.01, *p* = 0.97). DD rate (k) did neither statistically significantly correlate with impulsivity (*r* = 0.15, *p* = 0.35) nor with executive functions (*r* = −0.13, *p* = 0.45). There was also no statistically significant correlation between impulsivity and executive functions (*r* = −0.16, *p* = 0.34).

### Structural subnetwork associated with delay of gratification and delay discounting

In all the correlations reported below, the behavioral measure (DoG or DD) and the structural connectivity measure (number of streamlines) have been associated by partial correlation analyses while simultaneously controlling for age, sex, and intracranial volume, but also controlling for the mean translation and mean rotation parameters on a volume-by-volume basis derived from the realignment procedure (Yendiki et al., 2014). The alpha error probability (*p*-value), which is independent and different from the sensitivity/set *t*-value reported below, was set at *p* = 0.05 corrected for multiple comparisons using 5000 permutations of the DoG of DD ability across subjects.

In addition, we also checked whether the behavioral measures of interest (DoG and DD) are associated with motion during the acquisition of the DTI data to rule out the possibility that subjects showing enhanced self-control also moved less during scanning. The behavioral measures DoG and DD neither correlated statistically significantly with the mean translation parameters (*r* = 0.026, *p* = 0.87 and *r* = 0.040, *p* = 0.81, respectively) nor with the mean rotation parameters (*r* = −0.093, *p* = 0.57 and *r* = 0.038, *p* = 0.81).

#### Nighty-node structural whole-brain connectome analysis

For the 90-node structural whole-brain connectome analysis, a subnetwork with a more liberal set threshold as well as a subnetwork with a more conservative set threshold is reported. This set thresholds based on *t*-values are independent from the alpha error probability reported and therefore do not affect the false positive rate.

##### Delay of gratification

**Figure 1:**
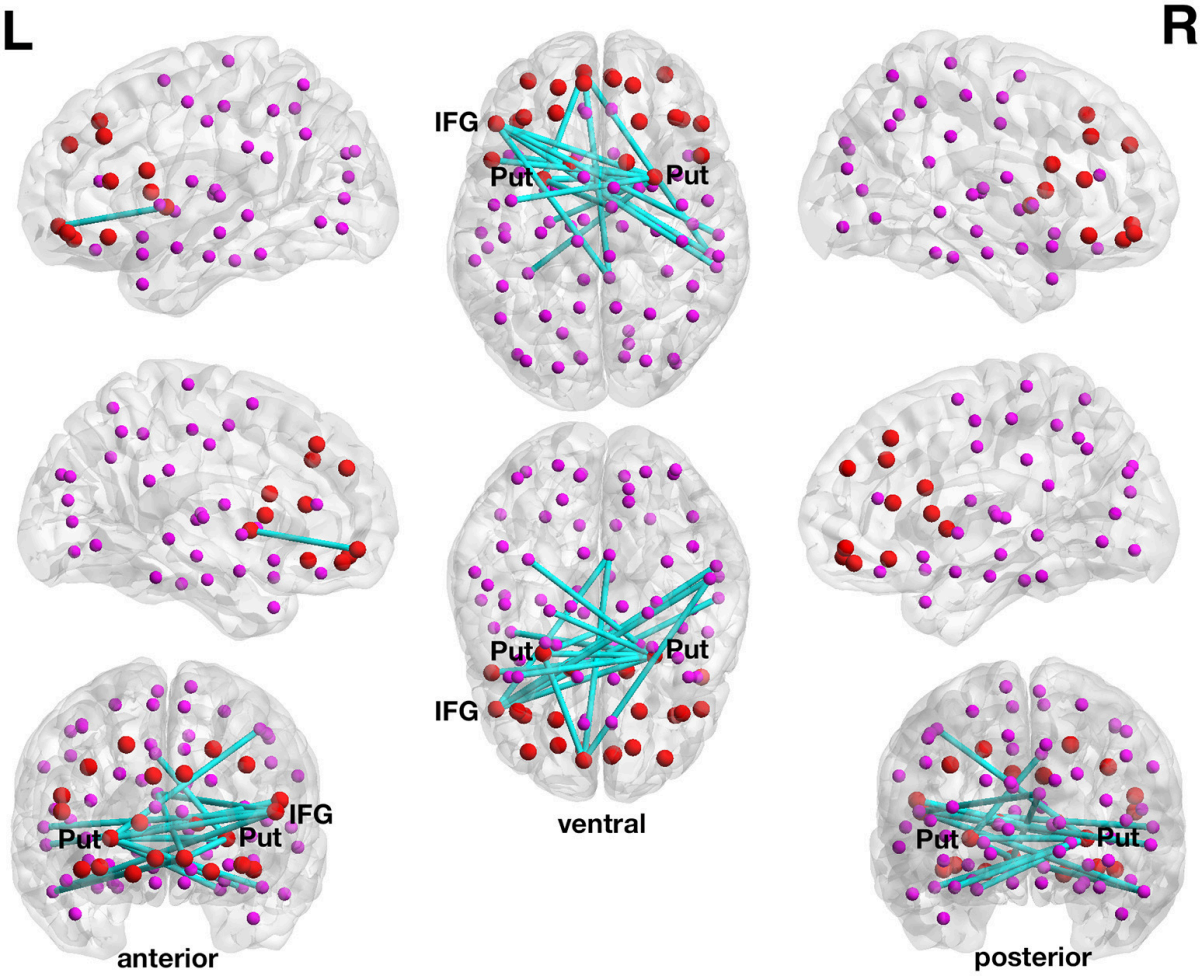
**Positive association between structural connectivity strength and delay of gratification in the 90-node whole-brain structural connectome analysis**. At a more liberal set (sensitivity) threshold (set *t*-value = 2.83, alpha error probability *p* = 0.048, corrected for multiple comparisons), a subnetwork showing 15 connections (turquoise lines) with positive correlations (0.422 ≤ *r* ≤ 0.530) between structural connectivity strength and delay of gratification performance has been found. These 15 connections were distributed over 14 nodes (larger red circles represent frontostriatal nodes, smaller pink circles represent all other nodes). The right putamen (7 connections), left frontal inferior gyrus (pars triangularis) (5 connections) as well as the left putamen (3 connections) serve as the most important hub regions within this structural subnetwork. A more conservatively thresholded network is presented in Supplementary Figure 1. The name of the nodes, the connections' *t*-values as well as the correlation coefficients of the associations can be found in Table 2. IFG, inferior frontal gyrus (pars triangularis); L, left; Put, putamen; R, right.

At a more liberal threshold, a subnetwork (set *t*-value = 2.83, *p* = 0.048, corrected) shows 15 edges with positive correlations (0.422 ≤ *r* ≤ 0.530) between structural connectivity strength and DoG performance and these 15 connections were distributed over 14 nodes (Figure 1, Table 2). Most of the subnetwork's connections are interhemispheric connections.

**Table 2:** **Positive correlations between structural connectivity strength and delay of gratification in the 90-node structural whole-brain connectome analysis**.

**Node**	**Node**	***t*-value (df = 33)**	**Correlation**
Frontal_Med_Orb_L	Temporal_Inf_R	3.59	0.530
**Frontal_Inf_Tri_L**	**Temporal_Mid_R**	**3.33**	**0.502**
**Putamen_L**	**Putamen_R**	**3.29**	**0.497**
**Frontal_Inf_Tri_L**	**Cingulum_Post_R**	**3.28**	**0.496**
**Temporal_Pole_Sup_L**	**Putamen_R**	**3.17**	**0.483**
**Frontal_Inf_Tri_L**	**Putamen_R**	**3.08**	**0.473**
Frontal_Med_Orb_L	Cingulum_Post_R	2.97	0.459
ParaHippocampal_L	Putamen_R	2.95	0.457
Frontal_Med_Orb_L	Putamen_L	2.94	0.456
Putamen_L	Cingulum_Mid_R	2.92	0.453
Frontal_Inf_Oper_L	Putamen_R	2.92	0.453
Fusiform_L	Putamen_R	2.90	0.451
Frontal_Inf_Tri_L	Temporal_Inf_R	2.88	0.448
Precentral_L	Putamen_R	2.84	0.443
Frontal_Inf_Tri_L	Temporal_Sup_R	2.83	0.442

*Connections printed in bold have been found with both set thresholds. The connection-wise t-values and correlation coefficients do not have associated p-values because alpha error probability has been controlled for the subnetwork as a whole. Ant, anterior; Inf, inferior; L, left; Med, medial; Mid, middle; Orb, orbital; Oper, opercular; Post, posterior; R, right; Sup, superior; Tri, triangular*.

The more conservatively thresholded subnetwork (set *t*-value = 3.07, *p* = 0.072, corrected) shows 5 edges with positive correlations (0.473 ≤ *r* ≤ 0.502) distributed over 6 nodes (Supplementary Figure 1, Table 2). However, this subnetwork showed only a trend (*p* = 0.072) toward significance.

The right putamen (7 connections), left pars triangularis of the left frontal inferior gyrus (5 connections) as well as the left putamen (3 connections) serve as the most important hub regions within the more liberal set thresholded structural subnetwork, i.e., the right putamen showed most connections with the effect of interest. The name of the nodes, the *t*-values of the connections as well as the correlation coefficients of the associations can be found in Table 2.

None of the connections showed an inverse correlation between DoG performance and connectivity strength.

##### Delay discounting

No statistically significant subnetworks have been found when the delay discounting rate has been correlated, neither negatively nor positively, with the strength of the structural connections in the 90-node whole-brain structural connectome analysis.

#### Twelve-node structural frontostriatal network analysis

For the 12-node structural frontostriatal network analysis, a subnetwork with a more liberal set threshold as well as a subnetwork with a more conservative set threshold is reported.

##### Delay of gratification

**Figure 2:**
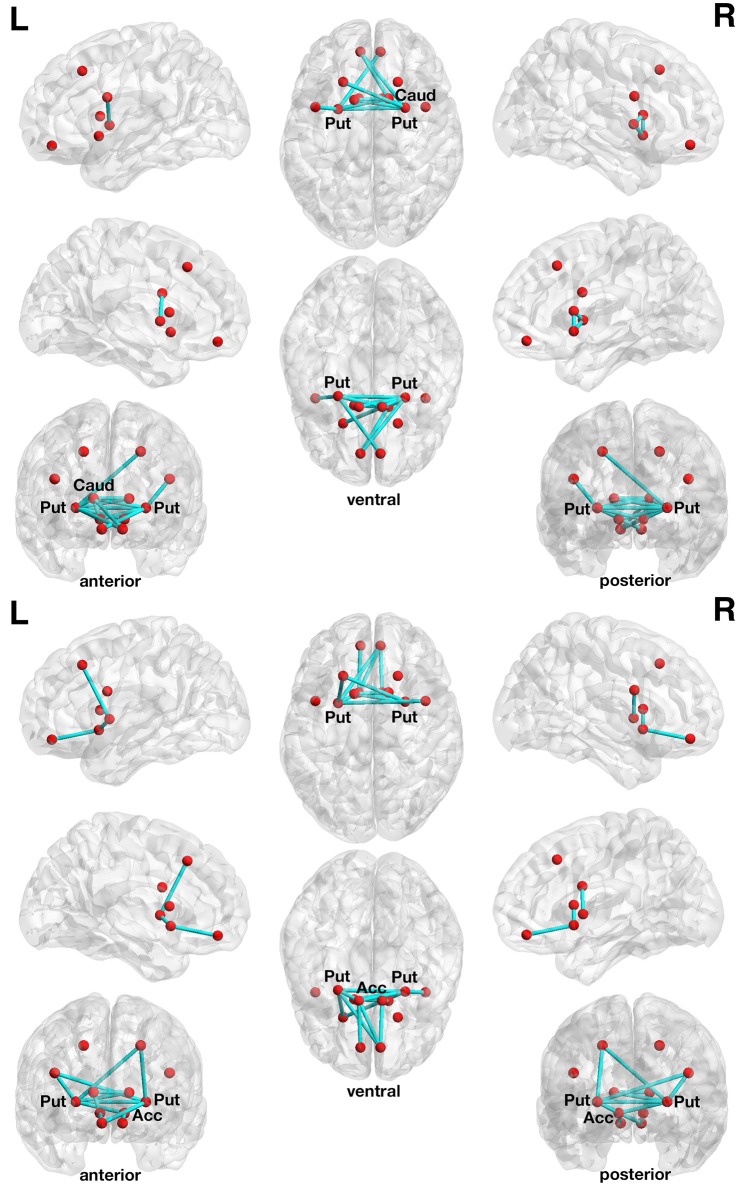
**Associations between structural connectivity strength and delay of gratification or delay discounting in the 12-node frontostriatal structural network analysis**. At a more liberal set (sensitivity) threshold (set *t*-value = 1.10, alpha error probability *p* = 0.013, corrected for multiple comparisons), a subnetwork showing 14 connections (turquoise lines) with positive correlations (0.196 ≤ *r* ≤ 0.443) between structural connectivity strength and delay of gratification performance has been found. These 14 connections were distributed over 10 nodes (red circles) **(upper panel)**. The right putamen (7 connections), left frontal inferior gyrus (pars triangularis) (5 connections) as well as the left putamen (3 connections) serve as the most important hub regions within this structural subnetwork. A more conservatively thresholded subnetwork is presented in Supplementary Figure 2 (upper panel). At a more liberal set threshold (set *t*-value = 0.85, alpha error probability *p* = 0.041, corrected), a subnetwork showing 14 connections (turquoise lines) with inverse correlations (−0.155 ≤ *r* ≤ −0.421) between structural connectivity strength and the delay discounting rate. These 14 connections were distributed over 10 nodes (red circles) **(lower panel)**. The left putamen (5 connections), right putamen (5 connections) and the left nucleus accumbens (4 connections) serve as important hub regions within this structural subnetwork. A more conservatively thresholded subnetwork is presented in the Supplementary Figure 2 (lower panel). Acc, nucleus accumbens; Caud, caudate nucleus; L, left; Put, putamen; R, right.

The more liberally thresholded subnetwork (set *t*-value = 1.10, *p* = 0.013, corrected) shows 14 connections with positive correlations (0.196 ≤ *r* ≤ 0.443) between structural connectivity strength and DoG performance. These 14 connections were distributed over 10 nodes (Figure 2, lower panel).

The more conservatively thresholded subnetwork (set *t*-value = 2.1, *p* = 0.004, corrected) shows 5 connections (0.349 ≤ *r* ≤ 0.443) distributed over 6 nodes (Supplementary Figure 2, upper panel). The name of the nodes, the connections' *t*-values as well as the correlation coefficients of the associations can be found in Table 3. The right putamen (7 connections), left putamen (5 connections), and the right caudate nucleus (5 connections) serve as important hub regions within these structural subnetworks. None of the connections showed an inverse correlation between DoG performance and structural connectivity strength.

**Table 3:** **Positive correlations between structural connectivity strength and delay of gratification in the 12-node structural frontostriatal network analysis**.

**Node**	**Node**	***t*-value (df = 33)**	**Correlation**
**Caudate_L**	**Putamen_R**	**2.84**	**0.443**
**Putamen_L**	**Putamen_R**	**2.71**	**0.427**
**Caudate_R**	**Accumbens_R**	**2.58**	**0.410**
**Putamen_L**	**OFC_R**	**2.50**	**0.399**
**Caudate_R**	**Putamen_L**	**2.14**	**0.349**
Putamen_R	Accumbens_L	1.89	0.313
Caudate_R	Putamen_R	1.85	0.307
Putamen_R	OFC_L	1.75	0.291
Caudate_L	Caudate_R	1.62	0.271
Putamen_L	VLPFC_L	1.52	0.256
Putamen_L	Accumbens_R	1.29	0.219
Putamen_R	Accumbens_R	1.29	0.219
Putamen_R	DLPFC_L	1.23	0.209
Caudate_R	OFC_L	1.15	0.196

*Connections printed in bold have been found in both network solutions (with both sensitivity/set thresholds). The connection-wise t-values and correlation coefficients do not have associated p-values because alpha error probability has been controlled for the subnetwork as a whole. DLPFC, dorsolateral prefrontal cortex; L, left; OFC, orbitofrontal cortex; R, right; VLPFC, verntrolateral prefrontal cortex*.

##### Delay discounting

With respect to delay discounting, a subnetwork with statistically significantly inverse correlations between the delay discounting rate and the strength of the structural connections has been found in the 12-node frontostriatal network analysis. Two different thresholded subnetwork are shown too. The more liberal thresholded subnetwork (set *t*-value = 0.85, *p* = 0.041, corrected) shows 14 edges with inverse correlations (−0.155 ≤ *r* ≤ −0.421) between structural connectivity strength and the delay discounting rate. These 14 connections were distributed over 10 nodes (Figure 2, lower panel).

The more conservative thresholded subnetwork (set *t*-value = 1.35, *p* = 0.049, corrected) shows 8 connections (−0.230 ≤ *r* ≤ −0.421) distributed over 8 nodes (Supplementary Figure 2, lower panel). The name of the nodes, the connections' *t*-values as well as the correlation coefficients of the associations can be found in Table 4. The left putamen (5 connections), right putamen (5 connections) and the left nucleus accumbens (4 connections) serve as important hub regions within these structural subnetworks. None of the connections showed a positive correlation between DD and structural connectivity strength.

**Table 4:** **Inverse correlations between structural connectivity strength and delay discounting in the 12-node structural frontostriatal network analysis**.

**Node**	**Node**	***t*-value (df = 33)**	**Correlation**
**Putamen_L**	**VLPFC_R**	**−2.67**	**−0.421**
**Putamen_L**	**OFC_R**	**−2.23**	**−0.362**
**Putamen_L**	**Putamen_R**	**−2.01**	**−0.330**
**Putamen_R**	**Accumbens_L**	**−1.79**	**−0.297**
**OFC_L**	**Accumbens_L**	**−1.75**	**−0.291**
**Putamen_L**	**Accumbens_L**	**−1.53**	**−0.257**
**OFC_R**	**Accumbens_R**	**−1.46**	**−0.246**
**Putamen_L**	**DLPFC_L**	**−1.36**	**−0.230**
Caudate_R	Accumbens_R	−1.34	−0.227
OFC_R	Accumbens_L	−1.33	−0.226
Putamen_R	DLPFC_L	−1.25	−0.213
Putamen_R	VLPFC_R	−1.09	−0.186
Caudate_L	Caudate_R	−0.90	−0.155
Caudate_L	Putamen_R	−0.90	−0.155

*Connections printed in bold have been found in both network solutions (with both sensitivity/set thresholds). The connection-wise t-values and correlation coefficients do not have associated p-values because alpha error probability has been controlled for the subnetwork as a whole. DLPFC, dorsolateral prefrontal cortex; L, left; OFC, orbitofrontal cortex; R, right; VLPFC, ventrolateral prefrontal cortex*.

### Functional subnetwork associated with delay of gratification and delay discounting

In all the correlations reported below, the behavioral (DoG or DD) and the functional connectivity measures have been associated by partial correlation analyses while simultaneously controlling for age, sex, and intracranial volume, but also controlling for the mean frame-wise displacement parameter according Power (Power et al., 2014). The alpha error probability (*p*-value), which is independent and different from the sensitivity/set *t*-value reported below, was set at *p* = 0.05 corrected for multiple comparisons using 5000 permutations of the DoG and DD ability across subjects.

In addition, we also checked whether the behavioral measures of interest (DoG and DD) are associated with motion during the acquisition of the rsfMRI data to rule out the possibility that subjects with better cognitive control also moved less during scanning. Neither DoG nor DD correlated statistically significantly with the mean frame-wise displacement parameter (*r* = −0.127, *p* = 0.44 and *r* = 0.223, *p* = 0.17, respectively).

#### Nighty-node functional whole-brain connectome analysis

For the 90-node functional whole-brain connectome analysis, a subnetwork with a more liberal set threshold as well as a subnetwork with a more conservative set threshold is reported.

##### Delay of gratification

In the 90-node functional whole-brain connectome analysis, there were no statistically significant associations, neither positive nor negative ones, between functional connectivity strength on one hand and DoG performance on the other hand.

##### Delay discounting

**Figure 3:**
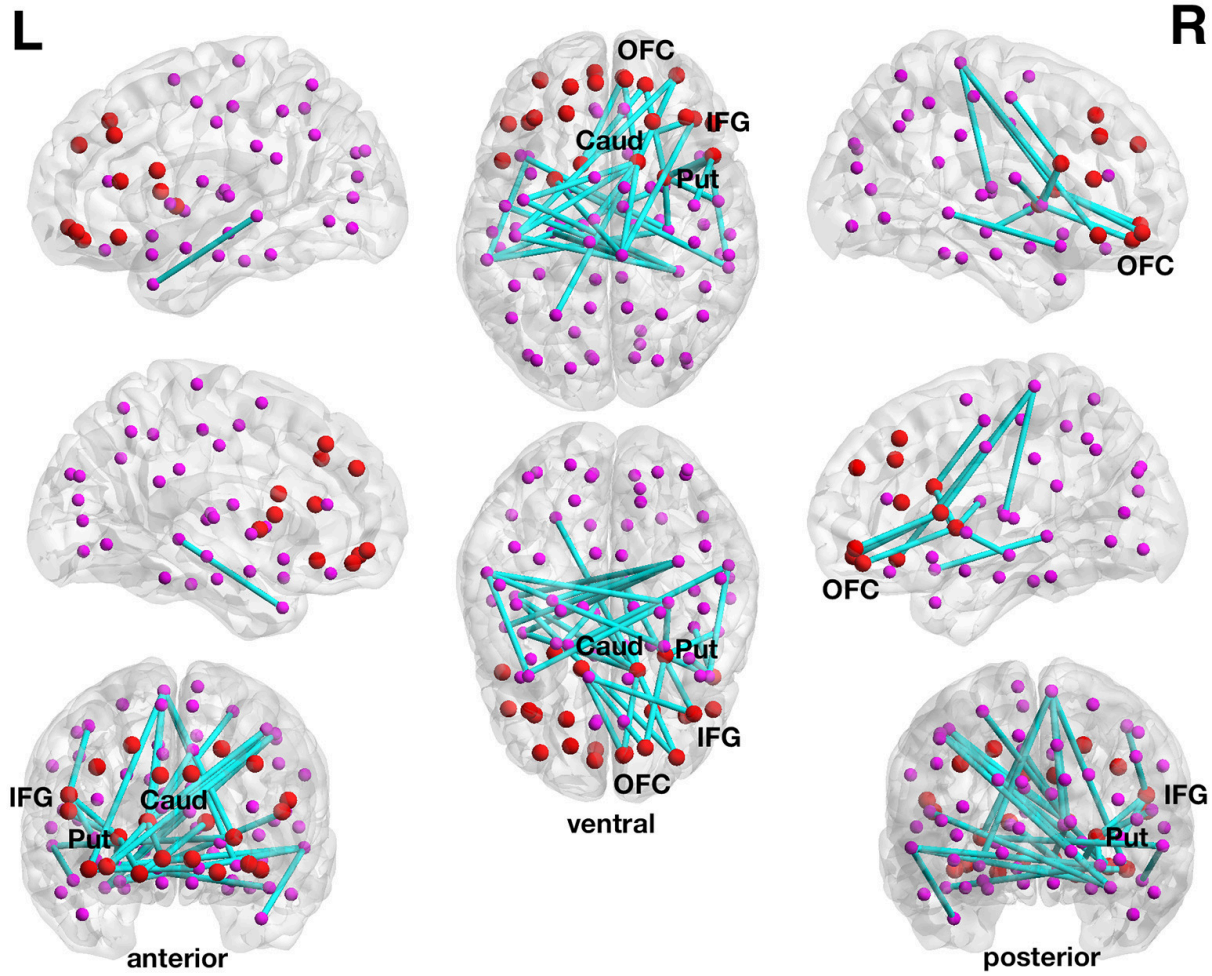
**Inverse association between functional connectivity strength and the delay discounting rate in the 90-node whole-brain functional connectome analysis**. At a more liberal set (sensitivity) threshold (set *t*-value = 3.87, alpha error probability *p* = 0.044, corrected for multiple comparisons), a subnetwork showing 30 connections (turquoise lines) with inverse correlations (−0.155 ≤ *r* ≤ −0.421) between functional connectivity strength and the delay discounting rate has been found. These 30 connections were distributed over 29 nodes (larger red circles represent frontostriatal nodes, smaller pink circles represent all other nodes). Although, the resulted subnetwork is rather large encompassing 30 connections, it is centered on frontostriatal nodes. Of the 30 connections, 20 connections have at least a prefrontal or a striatal node involved and 6 connections are direct frontostriatal connections. The subnetwork has 10 prefrontal nodes and 14 striatal nodes involved in these 30 connections. The right caudate nucleus encompasses 7 connections and the right putamen 4 connections and these two subcortical brain structures serve as striatal hub regions within this subnetwork. The right inferior frontal gyrus (pars opercularis) encompasses 3 connections and this cortical brain structure serves as a prefrontal hub region within this subnetwork. The name of the nodes, the connections' *t*-values as well as the correlation coefficients of the associations can be found in Table 5. Caud, caudate nucleus; IFG, inferior frontal gyrus (pars opercularis and orbitalis); L, left; Put, putamen; OFC, orbitofrontal cortex; R, right.

With respect to delay discounting, a subnetwork with statistically significantly inverse correlations between the delay discounting rate and the strength of the functional connections has been found in the 90-node functional whole-brain connectome analysis. The more liberally thresholded subnetwork (set *t*-value = 3.87, *p* = 0.044, corrected) shows 30 connections with inverse correlations (−0.155 ≤ *r* ≤ −0.421) between functional connectivity strength and the delay discounting rate. These 30 connections were distributed over 29 nodes (Figure 3).

Although, the resulted subnetwork is rather large encompassing 30 connections, it is centered on frontostriatal nodes. Of the 30 connections, 20 connections have at least a prefrontal or a striatal node involved and 6 connections are direct frontostriatal connections. The subnetwork has 10 prefrontal nodes and 14 striatal nodes involved in these 30 connections. The right caudate nucleus encompasses 7 connections and the right putamen 4 connections and these two subcortical brain structures serve as striatal hub regions within this subnetwork. The right inferior frontal gyrus (pars opercularis) encompasses 3 connections and this cortical brain structure serves as a prefrontal hub region within this subnetwork. There are also several orbitofrontal nodes in the right hemisphere that encompass more than only one connection. However, there are nodes other than the frontostriatal ones such as the right paracentral lobule and the right hippocampus that also serve as hub regions within this large subnetwork (see Table 5).

**Table 5:** **Inverse correlations between functional connectivity strength and delay discounting in the 90-node functional whole brain connectome analysis**.

**Node**	**Node**	***t*-value (df = 33)**	**Correlation**
**Cingulum_Mid_L**	**Caudate_R**^§§^	**−4.14**	−0.585
**Frontal_Inf_Oper_R**	**Putamen_R**^§^	**−3.98**	−0.569
**Precentral_R**	**Frontal_Inf_Oper_R**^§^	**−3.70**	−0.541
**Parietal_Sup_L**	**Caudate_R**^§§^	**−3.66**	−0.537
**Paracentral_Lobule_R**	**Caudate_R**^§§^	**−3.63**	−0.534
**Paracentral_Lobule_R**	**Putamen_L**^§§^	**−3.63**	−0.534
Hippocampus_R	Temporal_Mid_L	−3.59	−0.530
Amygdala_R	Temporal_Mid_L	−3.56	−0.527
**Frontal_Sup_Orb_R**	**Caudate_L**^§^	**−3.55**	−0.526
**Frontal_Inf_Oper_R**	**Insula_R**^§^	**−3.54**	−0.525
**Frontal_Med_Orb_R**	**Caudate_L**^§^	**−3.52**	−0.522
**Paracentral_Lobule_R**	**Thalamus_R**^§§^	**−3.52**	−0.522
**Frontal_Sup_Orb_R**	**Putamen_R**^§^	**−3.44**	−0.514
**Postcentral_L**	**Caudate_R**^§§^	**−3.41**	−0.510
Frontal_Inf_Orb_R	Paracentral_Lobule_R	−3.31	−0.499
Putamen_L	Temporal_Mid_R	−3.27	−0.495
Frontal_Mid_Orb_R	Caudate_R	−3.24	−0.491
Frontal_Inf_Orb_R	Olfactory_L	−3.23	−0.490
Hippocampus_R	Temporal_Pole_Sup_L	−3.21	−0.488
Hippocampus_L	Paracentral_Lobule_R	−3.20	−0.487
Precentral_L	Caudate_R	−3.19	−0.485
Rolandic_Oper_L	Fusiform_R	−3.17	−0.483
Precentral_L	Fusiform_R	−3.06	−0.470
Frontal_Med_Orb_R	Caudate_R	−3.00	−0.463
Temporal_Pole_Sup_R	Temporal_Mid_R	−2.95	−0.457
Fusiform_R	Postcentral_L	−2.94	−0.456
Rolandic_Oper_R	Putamen_R	−2.93	−0.454
Temporal_Mid_L	Temporal_Pole_Mid_L	−2.93	−0.454
Frontal_Mid_Orb_R	Postcentral_L	−2.90	−0.451
Hippocampus_R	Putamen_R	−2.89	−0.449

*Connections printed in bold have been found with both sensitivity/set thresholds. The subnetwork found with the more liberal threshold disintegrate at the more conservative threshold and the connections denoted with § are part of first component that is located frontostriatally, whereas connections denoted with §§ are part of the second component that is located parietostriatally. The connection-wise t-values and correlation coefficients do not have associated p-values because alpha error probability has been controlled for the subnetwork as a whole. Inf, inferior; Mid, middle; L, left; Oper, opercular; Orb, orbital; R, right; Sup, superior*.

At the more conservative threshold (set *t*-value = 3.40) the subnetwork shown above for the more liberal threshold disintegrated into two subsubnetworks or components. Both subsubnetworks consistent of 6 connections distributed over 7 nodes, but both showed only a statistical trend toward significance (alpha error *p* = 0.066, corrected). The first subsubnetwork with correlations between −0.514 and −0.569 is a frontostriatal component (Supplementary Figure 3, upper panel) that included the right putamen, left caudate nucleus, right inferior frontal gyrus (pars opercularis) and two orbitofrontal nodes, whereas the second subsubnetwork with correlations between −0.510 and −0.585 is a parietostriatal component (Supplementary Figure 3, lower panel) that included the right caudate nucleus, left putamen as well as the left superior parietal lobule, right paracentral lobule, and left postcentral gyrus. None of the connections of the 90-node whole-brain functional connectome analysis showed any statistically significant positive correlation between DD and functional connectivity strength.

#### Twelve-node functional frontostriatal network analysis

For the 12-node functional frontostriatal network analysis, a subnetwork with a more liberal set threshold as well as a subnetwork with a more conservative set threshold is reported.

##### Delay of gratification

In the 12-node functional frontostriatal network analysis, DoG performance did also not show any statistically significant association with functional connectivity strength.

##### Delay discounting

**Figure 4:**
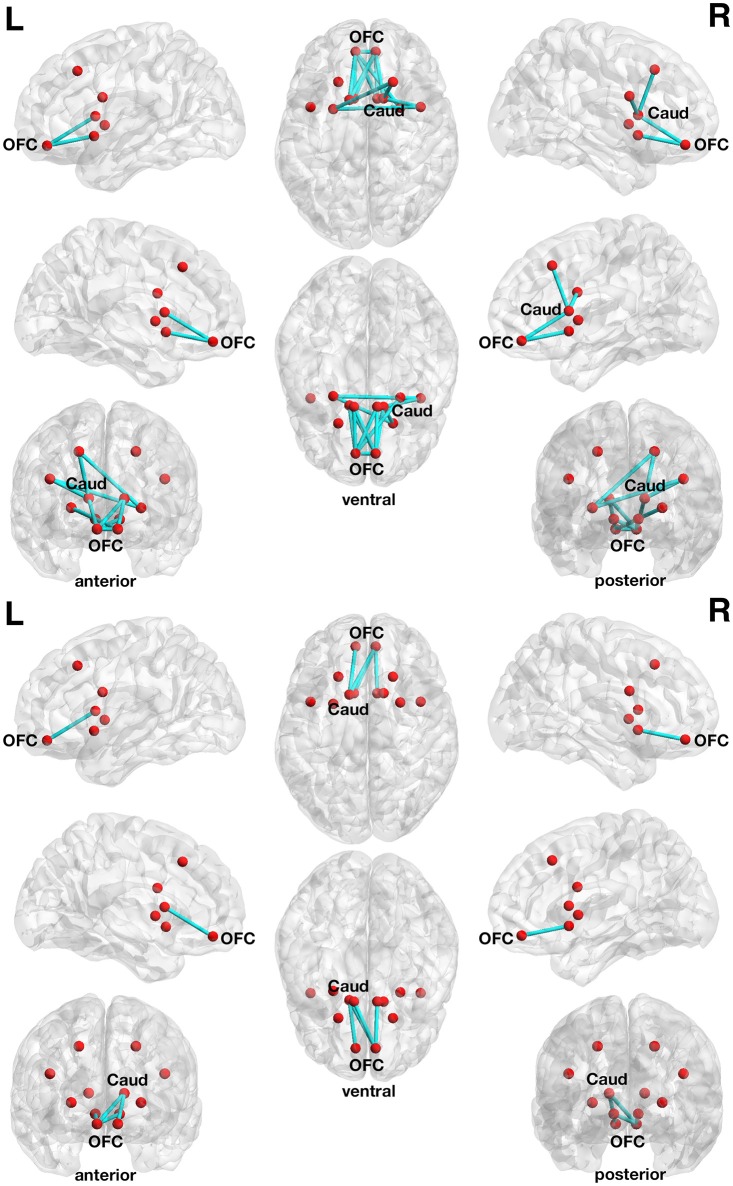
**Associations between functional connectivity strength and delay discounting in the 12-node frontostriatal functional network analysis**. At a more liberal threshold (set *t*-value = 1.65, alpha error *p* = 0.032, corrected for multiple comparisons), a subnetwork consistent of 13 connections with inverse correlations (−0.279 ≤ *r* ≤ −0.548) between functional connectivity strength and the delay discounting rate has been revealed. These 13 connections are distributed over 10 nodes (red circles represent frontostriatal nodes) **(upper panel)**. At a more conservative threshold (set *t*-value = 2.65, alpha error *p* = 0.015, corrected), a subnetwork consistent of 4 connections with inverse correlations (−0.442 ≤ *r* ≤ −0.480) distributed over 5 nodes has been revealed. The name of the nodes, the connections' *t*-values as well as the correlation coefficients of the associations can be found in Table 6. The right orbitofrontal cortex (5 connections) and the left orbitofrontal cortex (5 connections) together with the right caudate nucleus (3 connections) serve as hub regions within this subnetwork. None of the connections showed any statistically significantly positive correlation between delay discounting and functional connectivity strength. Caud, caudate nucleus; L, left; OFC, orbitofrontal cortex; R, right.

The correlation with delay discounting revealed an interesting subnetwork. At a more liberal threshold (set *t*-value = 1.65, alpha error *p* = 0.032, corrected for multiple comparisons), a subnetwork consistent of 13 connections with inverse correlations (−0.279 ≤ *r* ≤ −0.548) between functional connectivity strength and the delay discounting rate has been revealed. These 13 connections are distributed over 10 nodes (Figure 4, upper panel).

At a more conservative threshold (set *t*-value = 2.65, alpha error *p* = 0.015, corrected), a subnetwork consistent of 4 connections with inverse correlations (−0.442 ≤ *r* ≤ −0.480) distributed over 5 nodes has been revealed (Figure 4, lower panel). The name of the nodes, the connections' *t*-values as well as the correlation coefficients of the associations can be found in Table 6. The right orbitofrontal cortex (5 connections) and the left orbitofrontal cortex (5 connections) together with the right caudate nucleus (3 connections) serve as hub regions within this subnetwork. None of the connections showed any statistically significantly positive correlation between delay discounting and functional connectivity strength.

**Table 6:** **Inverse correlations between functional connectivity strength and delay discounting in the 12-node functional frontostriatal network analysis**.

**Node**	**Node**	***t*-value (df = 33)**	**Correlation**
Caudate_R	DLPFC_R	−3.76	−0.548
**Caudate_L**	**OFC_L**	**−3.14**	**−0.480**
**OFC_R**	**Accumbens_L**	**−2.95**	**−0.457**
**Caudate_L**	**OFC_R**	**−2.89**	**−0.449**
**OFC_R**	**Accumbens_R**	**−2.83**	**−0.442**
OFC_L	Accumbens_L	−2.63	−0.416
Putamen_L	DLPFC_R	−2.53	−0.403
Caudate_R	OFC_R	−2.33	−0.376
OFC_L	Accumbens_R	−2.18	−0.355
Putamen_R	OFC_L	−1.97	−0.324
Caudate_R	VLPFC_R	−1.97	−0.324
OFC_L	OFC_R	−1.89	−0.313
Putamen_L	VLPFC_R	−1.67	−0.279

*Connections printed in bold have been found in both network solutions (with both sensitivity/set thresholds). The connection-wise t-values and correlation coefficients do not have associated p-values because alpha error probability has been controlled for the subnetwork as a whole. DLPFC, dorsolateral prefrontal cortex; L, left; OFC, orbitofrontal cortex; R, right; VLPFC, verntrolateral prefrontal cortex*.

### Associations of structural and functional connectivity strength with impulsivity and executive functioning

We also correlated impulsivity and executive functioning with structural and functional connectivity strength using the same covariates of no interest as used for DoG and DD. The 90-node functional whole-brain connectome analysis and the 12-node functional frontostriatal network analysis did not reveal significant positive or inverse correlations between the functional connectivity strength of any connection of the connectivity matrix and impulsivity or executive functioning. The 90-node structural whole-brain connectome analysis revealed an inverse association between executive functioning and structural connectivity strength (set *t*-value: 4.50, alpha error *p* = 0.052) of only two connections involving only three nodes, although only on a trend level toward significance. One connection is located between the right middle occipital gyrus and the left putamen and the other connection is located between right middle occipital gyrus and the right middle cingulate cortex. No further statistical significant associations between structural connectivity and executive functioning have been found.

The 12-node structural frontostriatal network analysis revealed an inverse association between impulsivity and the structural connectivity strength (set *t*-value: 2.70, alpha error *p* = 0.018) of also only two connections involving only three nodes. One connection is located between the left nucleus accumbens and the right caudate nucleus and the other connection is located between nucleus accumbens and the orbitofrontal cortex. No further statistical significant associations between structural connectivity and impulsivity have been found.

## Discussion

In support of our hypothesis, we report for the first time that the strength of structural and functional frontostriatal connectivity predicts self-controlled behavior in healthy elderly subjects. In the present study, the concept of self-control (a.k.a. willpower) has been operationalized by DoG and DD paradigms, and behavioral and neuroimaging studies on DoG and DD in the elderly are of considerable relevance because it has been shown that high DoG (low DD) significantly predicts wellbeing in the healthy older adults (Forstmeier et al., 2011), a population that is rapidly growing in most countries all over the world.

In a previous study using the same study participants as in the current study, we sought to identify structural gray matter correlates of DoG and DD performance by using surface-based morphometry and subcortical segmentation procedures capable of measuring cortical thickness and surface area independently as well as subcortical volumes, respectively, based on T1-weighted MRI scans (Drobetz et al., 2014). That study revealed that DoG and DD performance are significantly associated with local cortical thickness and surface area in the DLPFC, VLPFC, OFC, and ACC on the one hand and with the volume of the caudate nucleus and nucleus accumbens on the other hand (Drobetz et al., 2014). The present findings corroborate the involvement of the above-mentioned prefrontal and striatal brain regions in self-controlled behavior and extent these gray matter findings by highlighting that not only is the integrity of the gray matter architecture of brain regions pivotal for self-control, but also the structural and functional connections among those DoG-relevant brain areas. From such a perspective, the present findings, together with those previously published (Drobetz et al., 2014), show strong convergence, although different MRI modalities (T1-weighted, DTI, and rsfMRI) and different computational neurostructural (cortical thickness, surface area, subcortical volumes, and structural connectivity) and neurofunctional analyses (seed-based functional connectivity) have been applied. In the following, we will first discuss in greater detail the associations between structural or functional connectivity strengths and self-controlled behavior as measured by DoG and DD. We will then relate our structural and functional findings to the literature by focusing on the frontostriatal brain regions (network nodes) associated with DoG and DD, followed by a discussion of our results with respect to the connectivity literature, focusing on the connections (network edges) associated with DoG and DD. We will then mention some limitations of the study and end with our conclusions.

Five years ago, Mischel et al. (2011) (among others) suggested that frontostriatal (and parietostriatal as well, although to a lesser extent) connectivity is important for DoG ability over an individual's lifespan. In line with this proposition, we empirically investigated and corroborated that the strength of frontostriatal fiber connections (structural connectivity) as well as the strength of frontostriatal communication (functional connectivity), as measured by different MRI techniques, are positively associated with DoG performance. Due to the inverse relationship between DoG and DD, the structural and functional frontostriatal connectivity strengths that were positively associated with the DoG ability were inversely correlated with the DD ability.

### Structural connections associated with delay of gratification and delay discounting

We used a whole-brain connectome (90-node network, excluding the cerebellum) approach as well as an approach focusing on specific frontostriatal regions (12-node network) in isolation from the rest of the brain. As can be seen in Figure 1 and Table 2, the whole-brain connectome approach highlights the specificity of our predicted findings; at the more liberal set threshold in the DoG structural 90-node network analysis, 15 out of 15 connections showing a positive correlation between structural connectivity strength and DoG performance involved either (pre)frontal or striatal brain regions. Considering the fact that the 90-node structural connectivity matrix theoretically contains 4005 possible connections, finding 15 connections with the effect of interest and all 15 connections are located in the predicted prefrontal and striatal regions makes the specificity of our findings even more remarkable.

As already mentioned, the right putamen serves as a hub region within this subnetwork and the left putamen also appeared in both differently thresholded subnetworks. The caudate nucleus did not show any structural connection with a statistically significant effect of interest, whereas the nucleus accumbens was not modeled as an individual node within the 90-node whole-brain connectome. In contrast, all striatal nodes (both putamina, both nuclei caudati, and both nuclei accumbi) of the 12-node network showed connections with positive associations between DoG performance and structural connectivity strength (see Figure 2, Table 3).

With respect to frontal regions, the medial OFC as well as those of the inferior frontal gyrus, i.e., the pars orbitalis, pars opercularis, and pars triangularis, were all involved in the subnetwork revealed by the structural whole-brain connectome analysis. In this 90-node network, however, the DLPFC and VLPFC were not represented in its full extent and in isolation (as a single node) and can hence not be labeled as such in the 90-node network. But these two regions were represented in the 90-node network in a distributed manner over the superior and middle frontal gyrus nodes with respect to the DLPFC and over the inferior frontal gyrus nodes with respect to the VLPFC meaning that the nodes containing DLPFC and VLPFC also containing other brain regions not part of these two functional areas. In contrast, the 12-node frontostriatal network analysis revealed that both OFC and the left VLPFC and left DLPFC possess connections that run to the putamen and caudate nucleus and show the effects of interest, although the size of the nodes of the 12-node network are smaller compared with the size of the nodes of the 90-node network, but contain only voxels belonging to these two functional regions.

Taken together, our structural network analyses revealed that mainly frontostriatal connections such as those connecting the putamen, caudate nucleus, and nucleus accumbens on the one hand with the DLPFC, VLPFC, and OFC on the other hand are associated with DoG and DD performance in healthy elderly individuals.

### Functional connections associated with delay of gratification and delay discounting

In our functional connectivity analyses, we also assessed whole-brain connections (90-node connectome analysis) as well as frontostriatal connections (12-node network analysis) in isolation from the rest of the brain. No functional subnetworks with statistically significant correlations between DoG and functional connection strength in the resting state were found in the 90-node functional whole-brain connectome analysis. However, the 90-node functional whole-brain connectome analysis revealed that the DD ability was statistically inversely correlated with functional connectivity strength in a 29-node subnetwork encompassing 29 mainly striatal and frontal nodes. The right caudate nucleus and the right putamen serve as striatal hub regions within this subnetwork. The right pars opercularis served as frontal hub.

In the 12-node functional frontostriatal network analysis, DoG performance did also not show any statistically significant association with functional connectivity strength. However, the 12-node functional frontostriatal network analysis revealed a subnetwork consistent of 13 connections with inverse correlations between functional connectivity strength and DD. As can be seen in Figure 4, Table 6, the right and left orbitofrontal cortex together with the right caudate nucleus serve as hub regions within this subnetwork.

Taken together, these findings show a large overlap between the structural and functional frontostriatal nodes, which each are positively correlated with DoG and/or inversely correlated with DD. The overlap is not so strong in the explicit connections found between the structural and functional network analyses, but is rather pronounced in the common nodes involved in DoG or DD ability. A recently published study in 120 healthy subjects (20–85 years old) applied whole-brain rsfMRI to measure functional connectivity and DTI to measure structural connectivity using automated fiber tractography of 18 major white matter tracts longitudinally over a time interval of 3.3 years mainly concluded that the anatomical alignment of structural connectivity alterations and functional ones seems restricted to specific networks and tracts and that in general changes in structural connectivity and those in functional connectivity are not necessarily strongly correlated (Fjell et al., 2017).

### Evidence for the frontostriatal network nodes associated with self-control

Here we report further evidence with respect to the network nodes (brain regions) whose structural and functional connectivity is associated with DoG and DD. As previously outlined (Drobetz et al., 2014), the DLPFC in general is a functionally defined brain area that is pivotal in the control of a variety of behavioral aspects, such as bottom-up and top-down control processes for working memory and attentional processes, the feeling of being present in a virtual reality (Jäncke et al., 2009), the regulation of emotions (Davidson et al., 2000), and even risky behaviors (Jäncke et al., 2008). In the context of self-controlled behavior, however, the DLPFC as well as the striatum are the key players in the processing of rewards and in decision making with respect to immediate and delayed rewards (Elliott et al., 2003; Wallis and Miller, 2003; McClure et al., 2004, 2007; Cho et al., 2010; Figner et al., 2010).

Evidence for the involvement of the VLPFC and ACC has been reported in the literature as well. The right VLPFC has been associated with behavioral inhibition (Cools et al., 2002) and bilateral VLPFC activity showed to be related to motor impulsivity as can be measured by go/no-go tasks (Goya-Maldonado et al., 2010), whereas activations in the left ACC have been demonstrated in response inhibition tasks (Horn et al., 2003), another important facet of DoG as shown in a rat experiment (Reynolds et al., 2002).

The left frontal pole/OFC has repeatedly been associated with impulsivity and its related constructs, including DD (McClure et al., 2004; Bjork et al., 2009; Matsuo et al., 2009) and has also been related to the value functions of rewards (Cohen and Ranganath, 2005). The importance of the OFC as a neural substrate in the decision to take delayed over immediate rewards and in subjective valuation has been reported using lesion studies in both animals (Cardinal et al., 2004; Winstanley et al., 2004; Rudebeck et al., 2006) and humans. Lesions to the medial OFC was associated with a significantly higher preference for an immediate over a delayed reward (Sellitto et al., 2010).

It has also been reported that the subjective value and magnitude of rewards are represented in the caudate nucleus (Delgado, 2007; Knutson et al., 2009), a critical component of the dopaminergic reward system (Breiter and Rosen, 1999), while the putamen seems to be activated in immediate and delayed rewards only in older participants (Samanez-Larkin et al., 2011). In a previous study by our group (Drobetz et al., 2014) using the same experimental subjects as investigated here, we found that DoG ability correlated positively with the volume of the left (*r* = 0.423, *p* = 0.004, one-tailed) and right (*r* = 0.300, *p* = 0.034, one-tailed) caudate nuclei, whereas DD was inversely correlated with the volume of the left (*r* = −0.273, *p* = 0.049, one-tailed) and right (*r* = −0.324, *p* = 0.024, one-tailed) caudate nucleus, as well as with the volume of the left nucleus accumbens (*r* = −0.321, *p* = 0.025, one-tailed; Drobetz et al., 2014).

### Evidence for the frontostriatal network connections associated with self-control

Recently published neuroimaging-based connectivity studies provide strong support for our findings. With respect to age-related structural connectivity in association with decision making, Samanez-Larkin and colleagues reported that decreased reward learning and decreased white matter integrity in specific pathways running from the thalamus to the medial PFC and from the medial PFC to the ventral striatum are associated with older age. This suggests that the integrity of frontostriatal white matter pathways critically supports reward learning (Samanez-Larkin et al., 2012). Peper et al. (2013) used DTI in young subjects and reported a link between the structural integrity of frontostriatal connections and DD performance. van den Bos et al. (2014) applied structural and functional MRI-based connectivity analyses and reported distinguishable striatal connections differentially related to DD. Structural and functional connectivity between the lateral prefrontal cortex and striatum was related to enhanced patience (corresponding to better DoG), whereas connectivity between the striatum and subcortical regions was related to enhanced impulsivity (corresponding to poorer DoG; van den Bos et al., 2014). The same group also investigated alterations in structural and functional connectivity of different frontostriatal fiber tracts during development. The authors showed that developmental increases in structural connectivity strength for connections reaching or originating from the right DLPFC were significantly associated with enhanced negative functional coupling (increased functional connectivity) with the striatum. An age-related decrease in discount rates was also reported (van den Bos et al., 2015).

In an fMRI study in children using a DoG paradigm, Luerssen and colleagues highlighted that a stronger attentional focus directed away from temptations was associated with enhanced functional coupling (increased functional connectivity) between the nucleus accumbens and several brain regions within the prefrontal and parietal cortices known to support self-control (Luerssen et al., 2015). In another fMRI study applying activity and effective connectivity analyses combined with an intertemporal monetary choice task, Hare and colleagues revealed a region in the left DLPFC (BA46) that showed increase activity in trials where subjects preferred delayed rewards. Furthermore, the strength of the functional connection between this region and a region in the VLPFC, commonly involved in the evaluation of stimulus values, enhanced at the time of the choice, mainly during trials where the subjects chose the delayed rewards (Hare et al., 2014).

A further fMRI study in adolescents and individuals in mid-adulthood used a hypothetical discounting task with monetary rewards delayed in the range of 1 week to 1 year and revealed that reductions in choice impulsivity (corresponding to enhanced DoG or reduced DD) with advancing age were related to alterations in brain activity in the VLPFC, ACC, ventral striatum, insula, inferior temporal gyrus, and in the posterior parietal cortex (Christakou et al., 2011). Even in nonhuman primates, there exists a strong link between frontostriatal connectivity strength and DoG ability. Using DTI, Latzman et al. (2015) investigated whether white matter tracts projecting from the prefrontal cortex to the caudate nucleus and the DoG ability are related in a sample of 49 captive chimpanzees (Pan troglodytes). After accounting for age, sex, and the time interval between the acquisition of the DTI data and the DoG assessment, enhanced white matter connectivity between the right dorsal prefrontal cortex and the caudate nucleus revealed to be significantly related to the acquisition (i.e., training) phase, but not related to the maintenance of the DoG ability. Further support for the association between DoG or DD on the one hand and structural and functional frontostriatal connectivity strength on the other has been reported elsewhere in the human and animal literature (Kim et al., 2012; Jimura et al., 2013; Li et al., 2013; Benningfield et al., 2014; Calluso et al., 2015) and therefore will not be reiterated in detail here.

## Limitations

Several limitations of the present study are worth mentioning. First of all, the findings presented here are correlative in nature and therefore do not necessarily imply any causal relationship between structural and functional connectivity strength on one hand and DoG and DD performance on the other hand. Second, it is not intuitive to assume that the strength of functional connectivity associated with DoG and DD during the resting state also accounts for differences in DoG and DD performance during active decision tasks involving immediate and delayed rewards. However, a recently published functional imaging study indicates that the brain's functional network architecture during task performance is shaped primarily by an intrinsic network architecture that is also present during the resting state (Cole et al., 2014). Third, while Mischel et al. (2011) have proposed that frontostriatal as well as parietostriatal connectivity is important for successful DoG performance, we did not construct or investigate specific parietostriatal networks in isolation from the rest of the brain, as was done for frontostriatal connectivity. However, our 90-node whole-brain structural or functional connectome analyses did not provide any evidence for the involvement of parietostriatal connections in DoG ability. Lastly, in order to establish the causality and dynamics of frontostriatal alterations as well as changes in DoG ability across lifespan, longitudinal studies are needed.

## Conclusions

Structural and functional connectivity strengths between striatum and OFC, DLPFC, and VLPFC show strong positive correlations with DoG ability and inverse associations with DD performance, suggesting that different brain regions and their connectivity within the frontostriatal network are crucial for self-controlled behavior, as measured using the DoG and DD paradigms specifically, and for willpower in general in the elderly population. The results of this study nicely correspond with the idea that the strength and integrity of frontostriatal connectivity are associated with self-control and willpower, not only in children and adolescences, but also in healthy elderly individuals. Since it has been shown that high DoG is a significant predictor of wellbeing in the older adults (Forstmeier et al., 2011), a population that is rapidly growing all over the world, neuroimaging studies on DoG in the healthy elderly are in our opinion of exceptional relevance for the society. Appropriate behavioral interventions aimed at enhancing self-control by means of enhancing frontostriatal connectivity might have the potential to enhance wellbeing in the elderly and might also reduce the costs of the health care systems.

## Ethics statement

This study was carried out in accordance with the recommendations of the cantonal ethical review board and approved by the ethics committee of the canton of Zurich, Switzerland, with written informed consent from all subjects. All subjects gave written informed consent in accordance with the Declaration of Helsinki. The protocol was approved by the ethics committee of the canton of Zurich, Switzerland.

## Author contributions

JH, CL, RD, SF, AM, and LJ designed and coordinated the study; JH, SF, AM and LJ supervised the study; JH and RD recruited the participants and acquired the behavioral and imaging data; JH and CL conducted the data preprocessing and analyses; HB contributed to network analysis; JH drafted the manuscript; AM and LJ critically evaluated the manuscript. All authors took part in results interpretation and discussion and gave final approval of the version to be published.

## Funding

This study was supported by the Swiss National Science Foundation (SNSF) and the Swiss Alzheimer Association with a grant to AM and SF (grant number 100014–124535) as well as by a Jacobs Foundation research grant to RD (no grant number available).

### Conflict of interest statement

The authors declare that the research was conducted in the absence of any commercial or financial relationships that could be construed as a potential conflict of interest. Especially, we do not have any financial interests in the Delay of Gratification Test for Adults (DoG-A), which is freely available upon request by author SF.
