# Supplementary material for: Strength of Structural and Functional Frontostriatal Connectivity Predicts Self-Control in the Healthy Elderly
Source: Front Aging Neurosci. 2016 Dec 23;8:307. doi: 10.3389/fnagi.2016.00307 (PMC5214688; doi:10.3389/fnagi.2016.00307)
Supplement: Supplementary file 1 [file DataSheet1.docx]

Supplementary Material

**Strength of Structural and Functional Frontostriatal Connectivity Predicts Self-Control in the Healthy Elderly**

Jürgen Hänggi^*^, Corinna Lohrey, Reinhard Drobetz, Hansruedi Baetschmann, Simon Forstmeier, Andreas Maercker & Lutz Jäncke

*** Correspondence:** Jürgen Hänggi: [j.haenggi@psychologie.uzh.ch](mailto:j.haenggi@psychologie.uzh.ch)

# Supplementary Figures

**1.1 Nighty-node whole-brain structural connectome analysis**


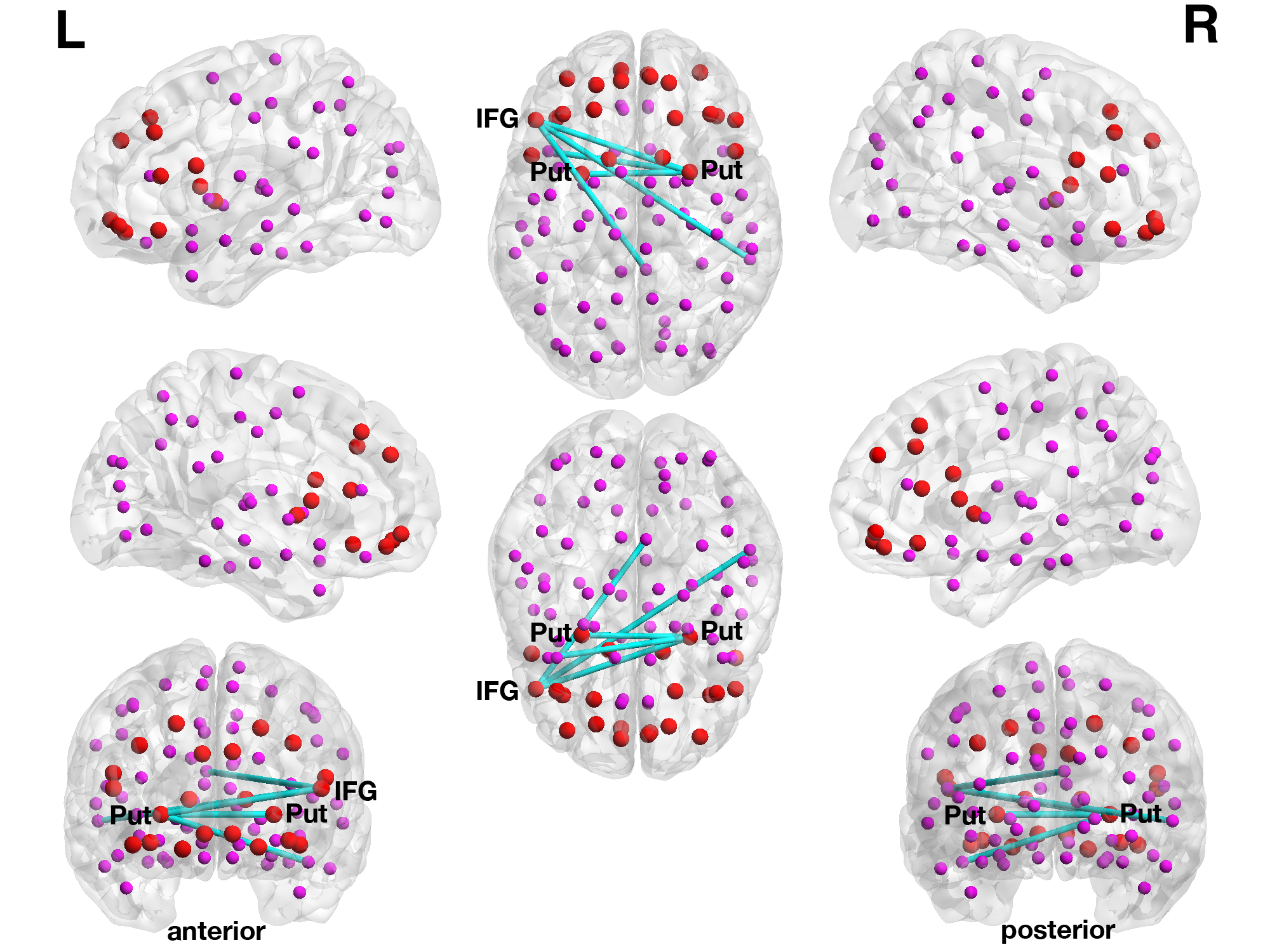


**Supplementary Figure 1. Positive association between structural connectivity strength and delay of gratification in the 90-node whole-brain structural connectome analysis.** At a more conservative set (sensitivity) threshold (set t-value = 3.07, p = 0.072, corrected for multiple comparisons), a subnetwork showing 5 connections (turquoise lines) with positive correlations (0.473 **≤** r **≤** 0.502) between structural connectivity strength and delay of gratification performance has been found. These 5 connections were distributed over 6 nodes (larger red circles represent frontostriatal nodes, smaller pink circles represent all other nodes). However, this subnetwork showed only a trend (p = 0.072) towards significance. A more liberally thresholded network is presented in Figure 1 in the main manuscript. The name of the nodes, the connections’ t-values as well as the correlation coefficients of the associations can be found in Table 2. An animation of the subnetwork presented in Supplementary Fig. 1 can be found in the Supplementary materials online (Supplementary animation 2). Abbreviations: IFG, inferior frontal gyrus (pars triangularis); L, left; Put, putamen; R, right.

**1.2 Twelve-node frontostriatal structural connectome analysis**


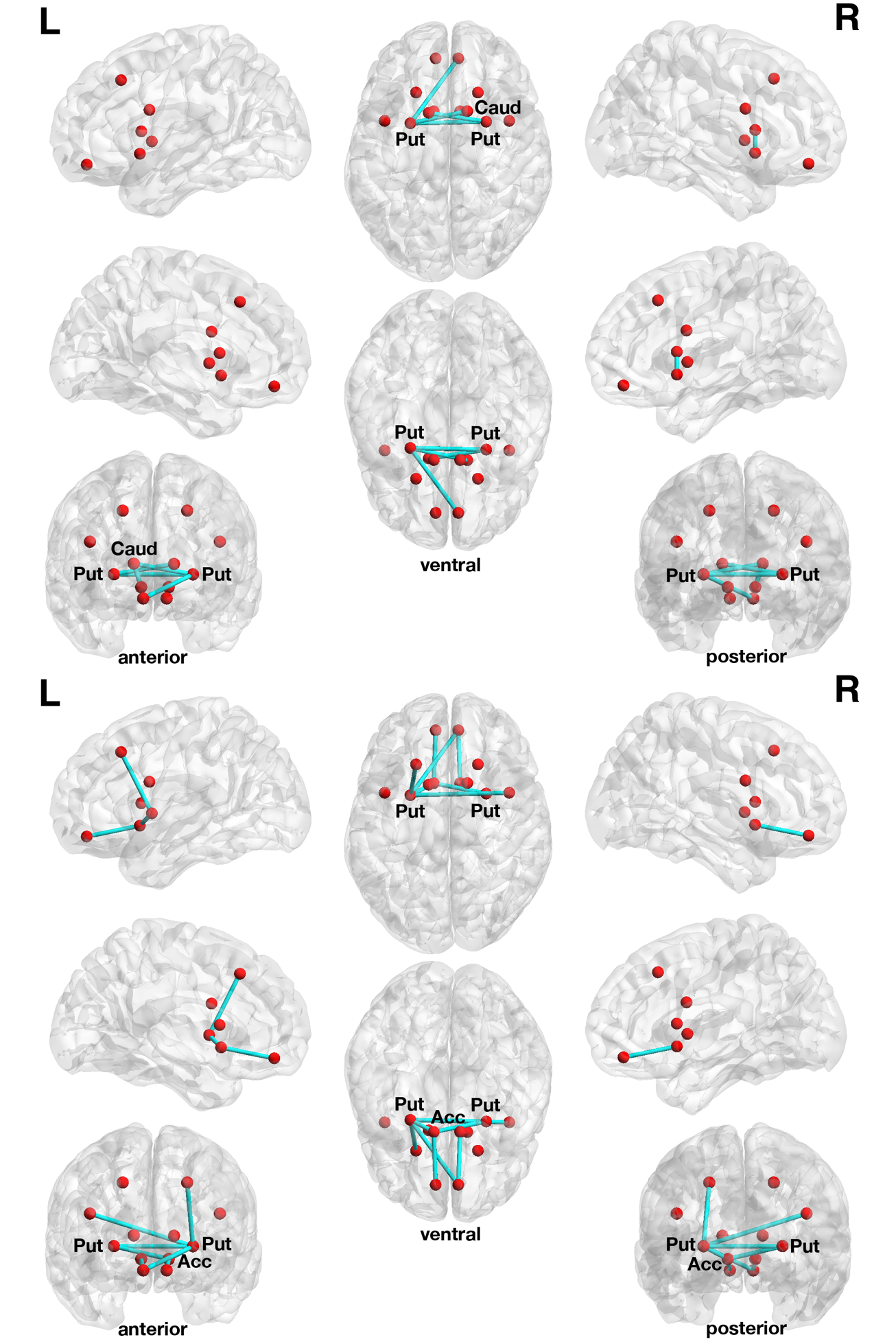


**Supplementary Figure 2.** **Associations between structural connectivity strength and delay of gratification or delay discounting in the 12-node frontostriatal network analysis.** At a more conservative set (sensitivity) threshold (set t-value = 2.10, alpha error probability p = 0.004, corrected for multiple comparisons), a subnetwork showing 5 connections (turquoise lines) with positive correlations (0.349 **≤** r **≤** 0.443) between structural connectivity strength and delay of gratification performance has been found. These 5 connections were distributed over 6 nodes (red circles) (upper panel). The right putamen (7 connections), left putamen (5 connections) and the right caudate nucleus (5 connections) serve as the most important hub regions within this structural subnetwork. A more liberally thresholded subnetwork is presented in Figure 2 (upper panel) in the main manuscript. At a more conservative set threshold (set t-value = 1.35, alpha error probability p = 0.049, corrected), a subnetwork showing 8 connections (turquoise lines) with inverse correlations (-0.230 **≤** r **≤** -0.421) between structural connectivity strength and the delay discounting rate. These 8 connections were distributed over 8 nodes (red circles) (lower panel). The left putamen (5 connections), right putamen (5 connections) and the left nucleus accumbens (4 connections) serve as important hub regions within this structural subnetwork. A more liberally thresholded subnetwork is presented in Figure 2 (lower panel) in the main manuscript. Abbreviations: Acc, nucleus accumbens; Caud, caudate nucleus; L, left; Put, putamen; R, right.

**1.3 Nighty-node whole-brain functional connectome analysis**


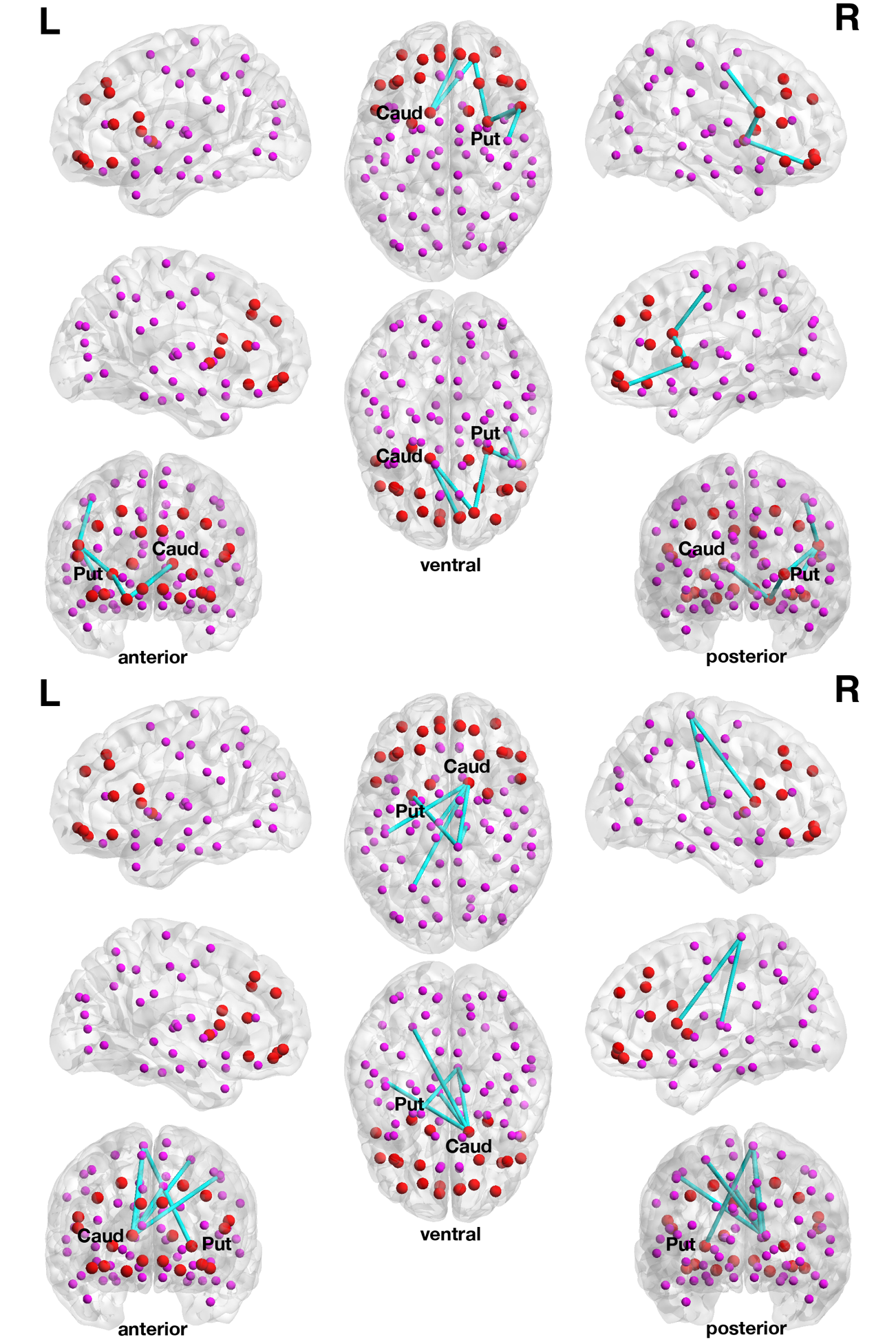


**Supplementary Figure 3.** **Associations between functional connectivity strength and delay discounting in the 90-node whole-brain functional connectome analysis.** At a more conservative set threshold (set t-value = 3.40) the liberally thresholded subnetwork shown in Fig. 3 in the main manuscript disintegrates into two components, a frontostriatal and a parietostriatal component. Both subnetworks consistent of 6 connections distributed over 7 nodes, but both showed only a statistical trend towards significance (alpha error p = 0.066, corrected for multiple comparisons). The first subnetwork with correlations between -0.514 **and** -0.569 is a frontostriatal component (upper panel) that included the right putamen, left caudate nucleus, right inferior frontal gyrus (pars opercularis) and two orbitofrontal nodes, whereas the second subnetwork with correlations between -0.510 **and** -0.585 is a parietostriatal component (lower panel) that included the right caudate nucleus, left putamen as well as the left superior parietal lobule, right paracentral lobule, and left postcentral gyrus. Larger red circles represent frontostriatal nodes, smaller pink circles represent all other nodes. A more liberally thresholded subnetwork is presented in Figure 3 in the main manuscript. Abbreviations: Caud, caudate nucleus; L, left; Put, putamen; R, right.
